# Nitrous Oxide Gas with Oxygen

**Published:** 1897-06

**Authors:** 


### Nitrous-Oxide Gas with Oxygen.

Nitrous-oxide gas with oxygen in removal of adenoids has the advantages that:

1. It is not attended with danger to life.
2. No preparations are required.
3. Hemorrhage is not affected by it.
4. Jactitation and cyanosis produced by pure nitrous oxide are absent.
5. Any position may be safely assumed.
6. Anæsthesia is longer than by the nitrous oxide alone.
7. Unpleasant after-effects are very rare.— *Gardner.*

